# Biventricular adaptation after patent ductus arteriosus ligation

**DOI:** 10.1038/s41390-025-04615-8

**Published:** 2025-11-21

**Authors:** Adrianne R. Bischoff, Patrick J. McNamara

**Affiliations:** 1 Division of Neonatology, Department of Pediatrics, University of Iowa, Iowa City, IA USA; 2 Department of Internal Medicine, University of Iowa, Iowa City, IA USA

## Abstract

Patent ductus arteriosus closure is followed by major adaptive changes in cardiac loading conditions, which may be associated with hemodynamic instability in some patients secondary to left ventricular systolic and diastolic dysfunction. Conventional echocardiography measures, including ejection fraction, have poor sensitivity for early detection of impaired left ventricular dysfunction. Speckle Tracking Echocardiography, which allows characterization of atrial and ventricular strain, may be an earlier and more sensitive marker of abnormal heart function. Recent guidelines suggest incorporation of speckle tracking echocardiography into multiparametric assessments of heart function in neonates. Its use may enhance clinical decision-making by improving early detection of dysfunction and enabling more accurate risk stratification following PDA closure.

Management of patent ductus arteriosus (PDA) closure in preterm infants remains one of the most controversial topics in neonatology with significant debate regarding the approach to treatment, particularly in the first 2 postnatal weeks.^1^ Irrespective of varying opinions regarding the need and/or the timing to intervene, the period after interventional closure often brings rapid shifts in loading conditions that can lead to hypotension, oxygenation/ventilation failure, or systemic hypertension.^2^ While most studies have traditionally focused on left ventricular (LV) adaptation and afterload changes, PDA physiology is fundamentally a multisystem, multi-chamber problem (Fig. 1a). In this issue of *Pediatric Research*, Toyoshima and colleagues take an important step forward by applying machine learning-based speckle-tracking echocardiography (STE) to capture not only LV, but also left atrium (LA) and right ventricular (RV) strain dynamics before and after PDA ligation, offering new insights into the cardiac adaptation that unfolds during this vulnerable window.^3^ This was a retrospective study including 32 preterm infants who underwent PDA surgery and 36 no-PDA controls. For the PDA group, transthoracic echocardiography was performed within 12 h before PDA ligation, 4–8 h and 24–48 h postoperatively. In the no-PDA group, a single echocardiography was obtained. Standard descriptive statistics as well as binary analyses were performed to compare preterm infants without a PDA and those undergoing PDA closure. Sequential data was analyzed using one-way analysis of variance with repeated measures. The authors conclude that there are early and differential recovery patterns after PDA ligation between LV, LA and RV function.

**Fig. 1:**
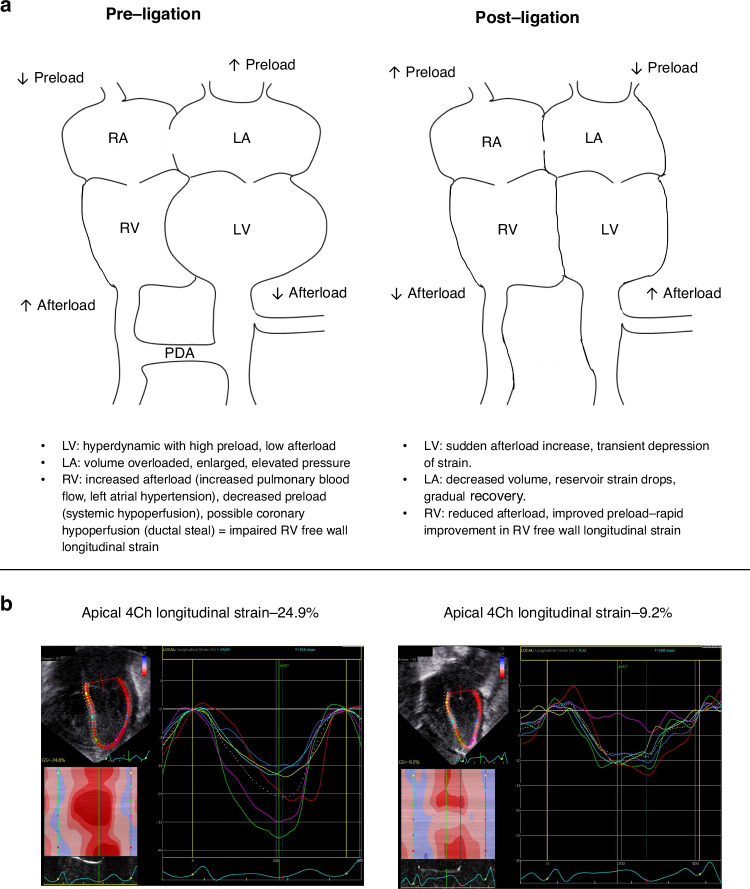
Changes in loading conditions before and after patent ductus arteriosus ligation. **a** The hemodynamic interplay of patent ductus arteriosus (PDA) across cardiac chambers. **b** Changes from normal longitudinal left ventricular strain to decreased strain after patent ductus arteriosus ligation. LV left ventricle, LA left atrium, RV right ventricle, RA right atrium, 4Ch four chamber.

Historically, PDA has been viewed primarily as a left heart problem - a disease of excessive preload and volume overload on the left atrium and ventricle.^4^ Preoperatively, the LV often demonstrates hyperdynamic function, with increased global longitudinal strain (GLS) - a surrogate of LV systolic performance - reflecting high preload and reduced afterload.^5^ Postoperatively there is also evidence of impaired LV diastolic function, which provides a plausible explanation for changes in lung recruitment secondary to pulmonary edema.^6^ Following surgical ligation, the abrupt removal of the low-resistance pulmonary circuit leads to a significant increase in afterload to the LV, corresponding with the well-described decline in LV GLS and the risk for systolic and diastolic dysfunction. Similar to Toyoshima et al., we have previously shown that LV GLS decreased from a mean of −20.6% pre- to −14.9% post-percutaneous PDA closure.^5^ However, the data from Toyoshima et al. adds important nuance by highlighting that the RV is not spared, a finding consistent with prior neonatal studies evaluating RV.^7–9^ Specifically, RV free wall strain (RV FWSL) is impaired in the preoperative period, possibly driven by a combination of LA hypertension, elevated pulmonary artery pressures, altered coronary perfusion and decreased preload from systemic hypoperfusion. Interestingly, they observed early postoperative improvement in RV strain, suggesting that decompression of the LA and decrease in flow-driven increased pulmonary pressures may lead to rapid right heart recovery after ligation.

The influence of prolonged exposure to high pulmonary blood flow on risk of LV dysfunction and post-procedural instability is poorly investigated. The impact may include changes in the pulmonary vasculature through remodeling and impact the net change in afterload experienced after ductal closure. Although post-ligation cardiac syndrome (PLCS) or post-transcatheter cardiorespiratory syndrome (PTCS) has been framed primarily because of LV afterload sensitivity, the contribution of the right heart has likely been underappreciated. Persistent flow-driven pulmonary hypertension may precondition the RV to tolerate post-ligation hemodynamic shifts, particularly in infants who develop systemic hypertension postoperatively. Furthermore, abnormalities in right coronary perfusion secondary to ductal steal may further impact RV tolerance. Understanding these chamber-specific adaptations may help refine how we stratify risk and optimize perioperative management.

These findings validate the need for an integrated, biventricular approach to hemodynamic monitoring. Susceptibility PLCS/PTCS to has been traditionally linked to myocardial immaturity, such that postnatal age at the time of ligation impacts risk.^10^ While PLCS is frequently described as a disease leading to hypotension and/or need for postoperative inotropes with or without respiratory compromise, PTCS appears to be more associated with respiratory symptoms in the setting of systemic hypertension.^2^ We hypothesize that the etiology of systemic hypertension after transcatheter PDA closure is multifactorial, and is specifically characterized by i) less need for sedatives and muscle relaxants which may promote vasodilation, ii) lack of mechanical confounders such as lung retraction and swings in intrathoracic pressure, and iii) possibly a difference in magnitude of inflammatory stimulus.^2^ It is possible that the endovascular procedure may trigger a more vasoconstrictor cascade versus the surgical approach. Importantly, the characteristic changes of impaired LV performance are less likely in older patients,^10^ which may relate to the less dramatic change in left heart loading conditions due to augmented pulmonary vascular resistance secondary to prolonged exposure of the immature pulmonary vascular bed to a high-volume PDA shunt.

Interestingly, despite the cohort’s high-risk profile – namely low gestational age, relatively early postnatal age, and procedural vulnerability - no infants in this study were reported to have developed PLCS.^3^ This finding invites reflection. Rather than suggesting selection bias, which is unlikely given the study’s comprehensive inclusion of virtually all PDA ligation cases at a single center, the absence of PLCS may be more plausibly attributed to differences in clinical definitions, timing of surgical intervention, or perioperative management strategies, including fluid and vasoactive support. These factors can significantly influence both the occurrence and recognition of PLCS/PTCS and merit further exploration.

Although the study^3^ reported relevant clinical data at discrete echocardiographic assessment points - including respiratory severity score, vasoactive-inotropic score, and use of catecholamines and vasodilators - a notable limitation remains in the absence of continuous or closely spaced serial data throughout the perioperative period. In particular, the reporting of inotrope use and respiratory severity score at only discrete time windows (e.g., 4–8 h and 24–48 h post-operatively) may miss dynamic fluctuations, especially within the 6–24-hour post-operative window, when most clinically significant oxygenation and hemodynamic deterioration typically occur. Therefore, while the authors clearly state that no patients met criteria for PLCS, the lack of detailed trend data limits the ability to assess for brief or subclinical episodes of instability. Acknowledging these nuances is essential to understanding how echocardiography abnormalities correlate with evolving clinical trajectories. These limitations highlight the importance of integrating serial clinical and hemodynamic data alongside advanced imaging findings to fully elucidate the clinical impact of echocardiography abnormalities in this population.

Data from Toyoshima et al. also provides additional insights regarding LA strain, a rarely assessed component in preterm PDA and ligation/closure studies, though a few studies have begun exploring LA strain as a marker of left heart loading in neonates.^11–13^ The LA may become maladapted from volume overload^5,12^ and delayed contractile recovery. Continued increased LA pressure, especially in the setting of LV dysfunction (whether systolic and/or diastolic), can increase the risk for the respiratory phenotype of PLCS, whereas LA hypertension contributes to secondary pulmonary edema and worsening oxygenation and ventilation.^4^ This may be even more pronounced in the setting of increased LV afterload and systemic hypertension, a phenomenon particularly observed following percutaneous PDA closure^2,5,14^ (Fig. 2). On the contrary, in infants with moderate-large interatrial communication (patent foramen ovale or atrium septum defect) the LA may be decompressed and partially mitigate the rise in LA pressure, albeit at the expense of an additional source of pulmonary overcirculation. Furthermore, LA strain may serve as an early marker for chronic shunt severity and remodeling.

**Fig. 2:**
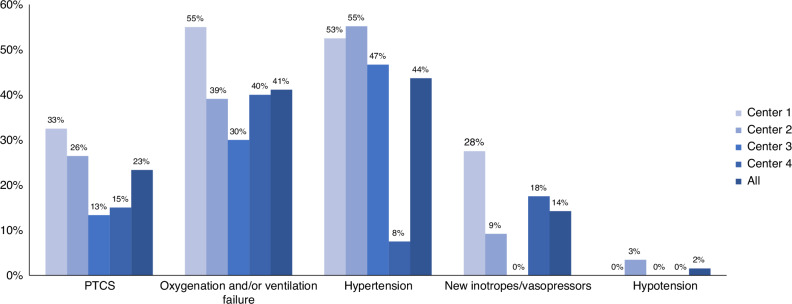
Clinical variability of post-transcatheter cardiorespiratory syndrome. Adapted from.^2^ PTCS Post-transcatheter cardiorespiratory syndrome.

The use of AI-assisted speckle-tracking technology in this study deserves particular attention. Ventricular strain assessment in neonates has historically been limited by technical challenges related to image quality, small cardiac size, and the expertise required for manual analysis.^15^ The machine learning-based AutoStrain platform allowed for rapid, fully automated contour detection and strain calculation across multiple chambers, with excellent reported intra- and inter-observer reproducibility. This level of automation holds clear appeal for clinical translation, particularly in the acute postoperative period when serial assessments may be valuable. There are, however, some important challenges that remain. Standardization across platforms, vendors, and strain algorithms is still lacking, limiting broader application and cross-study comparisons. In addition, LA strain remains difficult to capture reliably in the neonatal population. In one of our previous studies, even with experienced operators, phase-specific LA strain was not available for all eligible exams due to technical limitations.^5^ Without AI-assisted platforms, the ability to systematically measure individual LA strain components — reservoir, conduit, and contractile phases — has been largely impractical in routine neonatal practice. The use of automated strain platforms may help bridge this gap, offering new opportunities to integrate atrial mechanics into clinical decision-making.

The potential for strain analysis to contribute to perioperative risk stratification is particularly compelling. Traditional echocardiography measurements such as ejection fraction and fractional shortening do not change as significantly as longitudinal LV strain before and after PDA ligation/ closure^5,10,16^ (Fig. 1b). By characterizing chamber-specific adaptations, strain metrics may help predict which infants are most vulnerable to PLCS/PTCS and inform both prophylactic and therapeutic strategies. This approach is currently being evaluated in the MIDAS trial (Milrinone for the Prevention of Post-ligation Cardiac Syndrome), a randomized clinical trial designed to assess whether prophylactic milrinone reduces the incidence of PLCS and early mortality following PDA surgical or device closure.^17^ An ancillary MIDAS study is in development at select centers with targeted neonatal echocardiography (TNE) expertise, where serial echocardiographic data — including strain — will be collected longitudinally. This offers a unique opportunity to better characterize the evolving cardiovascular phenotype in infants who do or do not develop PLCS, and to examine how myocardial strain may respond to prophylactic therapies such as milrinone. While Toyoshima et al.‘s study was not powered to correlate strain parameters with clinical outcomes, it lays important groundwork for this next phase of investigation.

Although the application of strain analysis represents an important advance, the training and standardization of these assessment remains a significant barrier to widespread adoption in routine neonatal practice. Strain analysis requires not only high-quality image acquisition but also technical familiarity with vendor-specific algorithms, post-processing software, and chamber-specific nuances, particularly in neonates where small cardiac size and poor acoustic windows are common challenges. Current TNE training programs are increasingly incorporating strain into multiparametric assessment frameworks; however, there remains a steep learning curve, especially for atrial strain, where consistent capture of reservoir, conduit, and contractile phases is still limited even among experienced operators. As automated algorithms become more accessible, training efforts will need to evolve in parallel to ensure that strain assessment can be applied with both technical rigor and physiologic interpretation across diverse clinical settings.

Larger prospective, multicenter studies will be essential to validate the clinical utility of strain-guided risk assessment across broader PDA populations — including both surgical and catheter-based closures, different gestational and postnatal ages, treated and untreated infants, and extended longitudinal follow-up beyond the immediate 48-hour postoperative window. Importantly, future work should explore the predictive value of strain metrics not only for PLCS, but also for outcomes such as ventilation weaning, neurodevelopment, and specific phenotypes such as systemic hypertension or oxygenation failure, where LA strain may offer insight. The work by Toyoshima et al. adds meaningful physiologic data to the field, reinforcing the importance of comprehensive ventricular-atrial strain assessment in refining PDA management strategies. As the field of Neonatal Hemodynamics continues to evolve, the integration of novel imaging metrics such as AI-assisted strain analysis holds promise for advancing individualized, physiology-informed care in vulnerable populations.

Finally, strain assessment is also increasingly being incorporated into Neonatal Hemodynamics training programs as part of multiparametric TNE frameworks, complementing conventional measures of function, chamber size, and flow to provide a more sensitive, load-adjusted evaluation of myocardial performance. Compared to traditional parameters such as fractional shortening or ejection fraction, strain offers improved reproducibility, earlier detection of subclinical dysfunction, and greater sensitivity to load-dependent physiology — all of which are particularly relevant in the perioperative PDA setting. The most recent American Society of Echocardiography guidelines for Neonatal Targeted Echocardiography^18^ suggests that speckle-tracking echocardiography (STE) may serve as a valuable adjunct for assessing systolic performance, segmental abnormalities, and load-dependent changes; however, natural history data remain limited in neonates and further research is warranted to clarify its role in routine practice. Continued efforts to incorporate strain into both clinical care and structured training will be critical as we move toward more sophisticated, physiology-based management of preterm infants with PDA.
